# Use of Complementary and Alternative Medicine in the context of cancer; prevalence, reasons for use, disclosure, information received, risks and benefits reported by people with cancer in Norway 

**DOI:** 10.1186/s12906-022-03606-0

**Published:** 2022-07-29

**Authors:** Agnete E. Kristoffersen, Jorunn V. Nilsen, Trine Stub, Johanna Hök Nordberg, Barbara Wider, Dana Mora, Kiwumulo Nakandi, Mona Bjelland

**Affiliations:** 1 National Research Center in Complementary and Alternative Medicine (NAFKAM), Department of Community Medicine, UiT The Arctic University of Norway, Tromsø, Norway; 2 The Norwegian Cancer Society, Oslo, Norway; 3 Regional Cancer Center Stockholm Gotland, Stockholm, Sweden; 4 Dept Neurobiology, Care Sciences & Society, Division of Nursing & Dept Physiology & Pharmacology, Karolinska Institutet, Stockholm, Sweden

**Keywords:** Cancer, Self-management, Complementary therapies, Norway, Communication, Health information

## Abstract

**Background:**

Research exploring the use of specific Complementary and Alternative Medicine (CAM) modalities by Norwegian cancer patients is sparse. The aims of this study were therefor to map the different CAM modalities cancer patients use and further investigate their rationale for use, communication about use, self-reported benefits and harms, and their sources of information about the different modalities.

**Methods:**

In cooperation with the Norwegian Cancer Society (NCS), we conducted an online cross-sectional study among members of their user panel with present or previously cancer (*n* = 706). The study was carried out in September/October 2021 using a modified cancer-specific version of the International Questionnaire to Measure Use of Complementary and Alternative Medicine (I-CAM-Q). In total, 468 members, 315 women and 153 men, agreed to participate resulting in a response rate of 67.2%. The study was reported in accordance with the National Research Center in Complementary and Alternative Medicine’s (NAFKAM) model of reporting CAM use.

**Results:**

A large proportion of the participants (79%, *n* = 346) had used some form of CAM with a mean of 3.8 modalities each (range 1-17); 33% (*n* = 143) had seen a CAM provider, 52% (*n* = 230) had used natural remedies, while 58% (*n* = 253) had used self-help practices. Most of the participants used CAM to increase their quality of life, cope with the cancer disease or for relaxation/well-being (64%-94%), mostly with high satisfaction and low rates of adverse effects. Few used CAM to treat cancer or prevent it from spreading (16%, *n* = 55). The main information sources were health care providers (47%), the internet (47%), and family and friends (39%). More than half (59%) of the cancer patients discussed their use of at least one CAM modality with a physician.

**Conclusions:**

The results of this survey will provide health professionals with more in-depth insight into the patterns of CAM use by cancer patients and facilitate better-informed discussions with their patients. Considering the high use of CAM, reliable information provision supporting cancer care providers’ knowledge and health literacy among patients as well as good communication are crucial. The cooperation between the NCS and NAFKAM provides an example of how to address these issues.

## Background

In Norway approximately 35,000 people are diagnosed with cancer each year, more men (54%, *n* = 19,223) than women (46%, *n* = 16,292). Prostate (14%, *n* = 5,030), breast (10%, *n* = 3,424), lung (10%, *n* = 3,331), and colon cancer (9%, *n* = 3,121) are the most frequent cancer types in Norway. The median age at diagnosis is 70 years for both men and women. Due to early detection and new and more targeted treatment methods almost three out of four people survive their cancer today, and those who have cancer are living longer with their disease. The number of cancer survivors is increasing and at the end of 2020, there were 305,503 people alive who had previously been diagnosed with cancer [1].

Complementary and alternative medicine (CAM) is the term used for medicinal products and practices that are not part of standard medical care [2], and that are mainly offered outside the public health care system [3]. The term CAM generally covers modalities offered by providers, self-help practices, herbs and other natural remedies, special diets, physical activity, and spiritual practices. In Norway, visits to CAM providers, use of natural remedies (including herbs), and self-help practices represents what people broadly define as CAM [4]. The most commonly used CAM modalities in the general population in Norway are natural remedies (47%), followed by self-help practices (29%) and therapies received from CAM providers (15%) [5].

Previous studies demonstrated that 45% of Norwegian cancer patients use CAM within the first 5 years of their cancer diagnosis [6] and that annually 33.4% of all cancer patients use CAM [7]. However, we do not know more about the patterns of use, e.g. which therapies they use and for what purpose.

Female cancer patients who are young to middle-aged and highly educated have been described as the most frequent users of CAM in Norway and elsewhere [7–10]. Frequent use has also been reported among patients with symptoms related to their cancer, with metastatic disease; receiving palliative treatment; and diagnosed with cancer more than three months previously [11]. The most common reasons for use of CAM among cancer patients reported internationally are to increase the body's ability to fight the cancer, to improve physical and emotional well-being, provide hope, and to treat adverse effects as well as late and long-term effects from cancer and cancer treatment [12]. Patients experienced the best benefit from CAM for their physical and emotional well-being [12]. CAM can also be used as a coping strategy [13].

The most commonly used CAM modalities for cancer in Europe are intake of substances thought to have healing potential (homeopathy, herbal treatment, etc.) [14]. This is also the case in Norway where 18% of the cancer patients reported to have used “herbal or “natural” medicine” within a time frame of one year compared with 14% who had consulted CAM providers [7]. Most cancer patients in Norway use CAM in conjunction with conventional cancer treatment and use conventional health care services more frequently than cancer patients not using CAM [15].

Previous research shows that 65% of Norwegian hospitals offer some form of CAM as an add-on to conventional care [16]. Moreover, most oncology health care providers show a positive attitude towards CAM used to complement conventional cancer treatment [17, 18]. They also use these therapies themselves to some degree. A national multicenter survey of Norwegian health care providers working at oncology departments revealed that about 20% of the oncologists and 50% of the nurses used some sort of CAM [19]. However, a national survey from 2016 among oncology experts and CAM providers found that the majority of physicians and nurses also believed that combining complementary and conventional cancer treatment was associated with risks (78% and 93%, respectively); the percentage among CAM providers was markedly lower (43%) [18].

Patients with cancer highly value the input from health care providers about CAM [20–22]. Ideally, they should feel free to discuss all options without the fear of being rejected and/or stigmatized. This can best be achieved through open, transparent, non-judgmental, and informed discussions about possible outcomes of combining CAM and conventional treatment for cancer [23, 24]. However, only 18% of physicians and 26% of nurses working with cancer patients in Norway ask patients about their CAM use on a routine basis [23]. To increase the dialogue between oncology health care providers and patients about their use of CAM, there is a need for in-depth and nuanced knowledge of not only the prevalence but also the patterns of CAM use by cancer patients. To date, no research results have been published assessing the patterns of CAM use by cancer patients in Norway and this article aims to fill this gap.

### Aims of the study

The aims of this study were to map the different CAM modalities cancer patients use and further investigate their rationale for use, communication about use, self-reported benefits and harms, and their sources of information about the different modalities.

## Methods

In cooperation with the Norwegian Cancer Society (NCS), an online cross-sectional study was conducted among members of their user panel who currently have or previously have had cancer (*n* = 706). The study was carried out between 23rd September and 12th October 2021 using a modified, cancer-specific version of the International Questionnaire to Measure Use of Complementary and Alternative Medicine (I-CAM-Q) [25].

### Participants

The NCS’s user panel is a web panel of individuals with experience of cancer either as cancer patients or relatives of cancer patients including bereaved relatives. The panel consists of 906 people of which 706 people have cancer at present or have previously had cancer. The members are mostly women (75%) and more than half are between 50 and 69 years old. The members are recruited through the NCS’s webpage, social media, and a variety of social events.

All members of the NCS’s user panel, aged 18 years or above with a current or past cancer diagnosis were invited to participate in the survey. Members of the user panel who are relatives of someone who has, had, or died of cancer were excluded.

### Recruitment and data collection

**Fig. 1:**
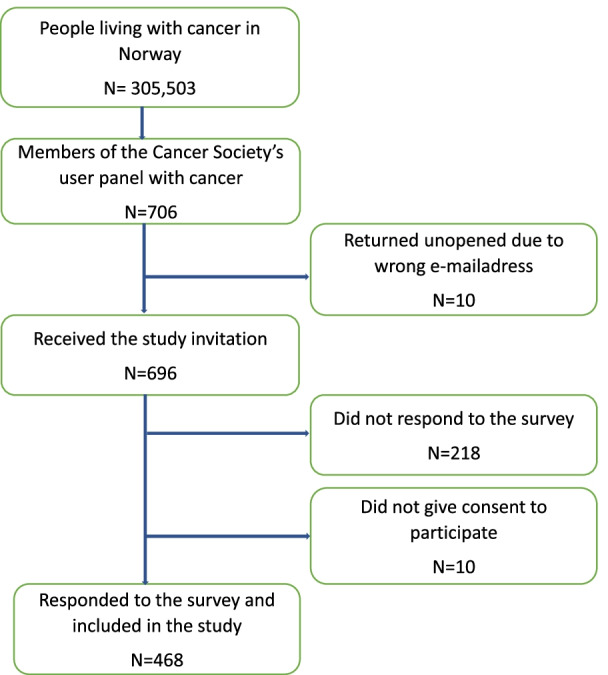
Flow chart of the included participants

Members of the panel fulfilling the inclusion criteria (*n* = 706) received a request by e-mail from the NCS with a link to the survey. The first page of the survey was an information letter where participants had to tick “agree to participate” in order to continue to the main survey. The survey was distributed online only. A total of 10 e-mails were returned as undeliverable leading to 696 members of the NCS’s user panel receiving the invitation. A total of 478 members responded. However, ten did not give their consent to participate and were excluded from the study. Consequently, 468 agreed to participate resulting in a response rate of 67.2% (Fig. 1).

### Measures

To compare CAM use across different studies, the National Research Center in Complementary and Alternative Medicine (NAFKAM) in Norway developed the NAFKAM model of reporting CAM [4]. In the model CAM activities were categorized in six different levels; CAM level one represents more than three visits to one or more CAM providers (not collected in the current study); CAM level 2 represents one or more visits to CAM providers; CAM level 3 represents CAM level 2 and/or use of natural remedies and/or self-help practices; CAM level 4 represents CAM level 3 and/or use of special diets; CAM level 5 represents CAM level 4 and/or use of physical activity, while CAM level 6 represents CAM level 5 and/or use of spiritual practices [4].

The I-CAM-Q was developed according to the NAFKAM model for classifying the use of CAM [25] and included visits to CAM providers, natural remedies, self-help practices, dietary supplements, special diets, physical activity, and spiritual practices (see Tables 2, 3, 4, 5, 6 and 7 for the specific modalities asked in this particular study). Socio-demographic data such as income and education were also collected. Data on age, gender, and cancer diagnosis had already been collected by the NCS for all members when they signed up to the user panel and were added to the survey questions for all participants. For all modalities used, the participants were asked follow-up questions about the reason(s) for CAM use ((1) To treat/slow down the cancer or prevent the cancer from spreading; (2) Treat adverse effects / late and long-term effects of the cancer or cancer treatment; (3) Strengthen the body / immune system; (4) Increase quality of life, coping, relaxation or well-being; (5) Other reasons), and possible adverse effects ((1) Yes, serious; (2) Yes, moderate; (3) Yes, mild; (4) No; (5) Do not know). According to the type of CAM (i.e. CAM provider; natural remedies; self-help practices; special diets; physical activity; and spiritual practice), the participants were asked how they experienced the possible effects of the modalities, with the following options: (1) Experienced that I got better; (2) No change; (3) Got worse; and (4) Do not know. In addition, they were asked where they gathered the information about the modality/approach with the following response categories: (1) Internet / media; (2) Health care providers (doctor / nurse etc.); (3) CAM provider; (4) Friends, family etc.; (5) Other; (6) Do not remember / do not know; (7) Did not receive / did not seek information, and further whether they had discussed this use of treatments with their: (1) General Practitioner (GP); (2) Oncologist; (3) Nurse; (4) Other health care providers (nutritionist etc.); (5) CAM provider; (6) None of these; (7) Do not remember / do not know.

### NAFKAM’s model of reporting CAM

The NAFKAM’s model of reporting CAM is a six-level model describing the extent of utilization of CAM with six cut-off points that would represent widely accepted levels of exposure to CAM, where the next levels in the model always include the previous levels (see Table 8 for a visual description of the model) [4]. The study was reported in accordance with NAFKAM’s model [4] of reporting use of CAM since diagnosis among cancer patients at level 2–6. Data on CAM level 1 (more than three visits to CAM providers) could not be reported as number of visits were not specified. As CAM at levels 2–3 is what mostly is considered as CAM in Norway, the associations for CAM use are presented for CAM level 2 (visits to CAM providers) and level 3 (visits to CAM providers and/or use of natural remedies, and/or self-help practices) only. Data on dietary changes and the use of vitamins and minerals were also collected and will be presented in a separate paper.

### Measures of personal characteristics

Age was obtained as an open question and assessed as a continuous variable as well as categorical after being merged into the following groups; *19–50 years*; *51–64 years,* and *65 years *or* more*.

Level of education was collected using four categories: (1) Primary school up to 10 years’ duration; (2) Secondary school 10–12 years’ duration; (3) College/university less than 4 years’ duration; and (4) College/university 4 years’ duration or more.

Household income was collected using the following categories NOK < 400,000 (low income); NOK 400,000–799,000 (medium income), and NOK 800,000 or more (high income) in addition to an option not to provide income information.

Other personal characteristics included sex (female, male) and place of residence (merged into the Norwegian regions South-East, South, West, Mid (Trøndelag), and North).

### Statistics/ power calculation

With a margin of error of 5%, a confidence level of 95%, and a heterogeneity of 50%, we needed a minimum sample of *n* = 384 to represent the Norwegian cancer population of 305,503 for adequate study power [26]. Descriptive statistics were carried out using Cross-tabulation and frequency analyses. For between-group analyses, Pearson chi-square tests and Fisher exact tests were used for categorical variables and binary logistic regression for adjusted values. For continuous variables, independent sample t-tests were used. Significance levels were set at *p* < 0.05. The analyses were conducted using SPSS V.28.0 for Windows.

## Results

The members of the NCS’s user panel consist of more women (75%) than men (25%) resulting in more women than men in the study (67% and 33%, *p* < 0.001) with a mean age of 57.3 and 62.9 years respectively (*p* < 0.001). The majority of participants had college or university education (63%), high income (46%), and were living in the South-Eastern part of Norway (52%). Most of the participants lived with a spouse/partner (67%); however, more men (75%) than women (63%, *p* = 0.008, Table 1).

**Table 1:** Basic characteristics of the participants and associations of CAM use

	**Total**		**Women**		**Men**			**CAM level 2**^**1**^			**CAM level 3 **^**2**^		
	**%**	***n* = 468**	**%**	***n* = 315**	**%**	***n* = 153**		**%**	***n* = 143**	***p*-value**	**%**	***n* = 346**	***p*-value**
**Sex**							< 0.001*			< 0.001*			0.002*
Women	67.3	315						38.9	114		83.1	246	
Men	32.7	153						20.3	29		69.9	100	
**Age**							< 0.001*			0.043*			0.735*
19–50 years	23.1	100	27.9	81	13.3	19		35.0	35		81.0	81	
51–64 years	41.3	179	43.4	126	37.1	53		38.0	68		77.1	138	
65 years or more	35.6	154	28.6	83	49.7	71		25.3	39		79.2	122	
Mean age (SD)	59.2 (11.295)		57.3 (11.277)		62.9 (10.408)		< 0.001’	57.36 (10.713)		0.019’	59.0 (11.451)		0.511’
**Education**							0.319*			0.003*			< 0.001*
Primary (less than 10 years)	6.5	28	5.2	15	9.1	13		3.6	1		46.4	13	
Secondary (10–12 years)	28.0	131	29.3	85	32.2	46		32.8	43		80.9	106	
University less than 4 years	33.9	147	35.9	104	30.1	43		39.5	58		81.0	119	
University 4 years or more	29.3	127	29.7	86	28.7	41		31.5	40		81.1	103	
**Household income**							0.477*			0.242*			0.074*
Low (less than NOK 400,000)	10.4	45	10.4	45	10.3	30		10.5	15		73.3	33	
Middle (NOK 400,000 – 799,000)	35.1	152	35.1	152	35.9	104		33.6	48		73.0	111	
High (NOK 800,000 or more)	46.4	201	46.4	201	44.5	129		50.3	72		83.6	168	
Did not reply	8.1	35	8.1	35	9.3	27		5.6	8		82.9	29	
Household**													
Live alone	20.7	97	22.9	72	16.3	25	0.103*	36.1	35	0.435*	75.3	73	0.331*
Live with a partner	66.9	313	62.9	198	75.2	115	0.008*	32.3	101	0.707*	80.2	251	0.266*
Live with own children	18.2	85	21.3	67	11.8	18	0.012*	36.5	31	0.441*	85.9	73	0.076*
Other	1.5	7	1.6	5	1.3	2	1.000^	14.3	1	0.435^	85.7	6	1.000^
**Place of residence (region)**							0.460*			0.497*			0.737*
South-East	51.7	242	53.3	168	48.4	74		30.6	71		78.9	183	
South	4.3	20	4.1	13	4.6	7		40.0	8		85.0	17	
West	24.8	116	22.5	71	29.4	45		30.5	32		75.7	81	
Mid (Trøndelag)	8.5	40	8.3	26	9.2	14		41.2	14		77.1	27	
North	10.7	50	11.7	37	8.5	13		40.0	18		84.4	37	

*Pearson chi-square test; ^Fisher exact test; ‘Independent sample t-test; ^1^ CAM level 2: One or more visits to CAM providers; ^2^ CAM level 3: One or more visits to CAM providers, use of CAM natural remedies and/or CAM self-help practices; **Multiple choice

More than half of the women suffered from breast cancer (58%) followed by female genitalia cancer (12%) and gastrointestinal cancer (11%). Men, on the other hand, were mostly diagnosed with male genitalia cancer (34%) followed by gastrointestinal cancer (20%) and lymphoma (14%). About a third of the participants (34%) were in active cancer treatment at the time of the survey (Table 2). A total of 12% had cancer at more than one site.

### Associations for CAM use

The clearest indicator for the use of CAM was female gender as women were significantly more likely to use CAM than men, 39% vs 20% (CAM level 2) and 83% vs 70% (CAM level 3, *p* < 0.003). Participants with the lowest level of education (primary school) were less likely to use CAM (*p* < 0.004, Table 1). Those visiting CAM providers (CAM level 2) were more likely to be middle-aged (51–64 years, *p* = 0.043, Table 1). Both breast cancer and skin cancer were indicators for high use of CAM; however, not when adjusted for gender. That was also true for male genital cancer that indicated low use of CAM (Table 2).

**Table 2:** Cancer-related characteristics of the participants and associations of CAM use

	**Total**		**Women**		**Men**			**CAM level 2**^**1**^			**CAM level 3 **^**2**^		
	**%**	***n *= 468**	**%**	***n* = 315**	**%**	***n* = 153**		**%**	***n* = 143**	***p*-value**	**%**	***n* = 346**	***p*-value**
Cancer site**													
Breast	39.1	183	57.8	172	0.7	1	< 0.001*	42.0	71	0.001*	80.7	138	0.474*
Gastrointestinal	13.7	64	10.5	33	20.3	31	0.004*	22.6	14	0.064*	79.0	49	0.964*
Male genitalia	11.1	52	0.0	0	34.0	52	< 0.001*	18.2	9	0.028*	62.5	30	0.003*
Lymphoma	8.8	41	6.3	20	13.7	21	0.008*	25.6	10	0.318*	74.4	29	0.537*
Female genitalia	8.1	38	12.1	38	0.0	0	< 0.001*	41.7	15	0.237*	91.7	33	0.049*
Malignant melanoma	4.7	22	4.4	14	5.2	8	0.707*	27.3	6	0.571*	72.7	16	0.433^
Head and neck	3.8	18	1.6	5	8.5	16	< 0.001*	23.5	4	0.448*	82.4	14	1.000^
Lung	3.2	15	2.5	8	4.7	7	0.268^	26.7	4	1.000^	78.6	11	1.000^
Sarcoma	3.0	14	3.8	12	1.3	2	0.160^	35.7	5	0.779^	85.7	12	0.744^
Skin	2.4	11	2.5	8	2.0	3	1.000^	20.0	2	0.509*	50.0	5	0.039^
Leukemia	2.4	11	2.2	7	2.6	4	0.755^	27.3	3	1.000^	72.7	8	0.707^
Bone marrow	2.1	10	1.9	6	2.6	4	0.735^	50.0	5	0.308^	100	10	0.129^
Brain tumor	1.9	9	0.6	2	4.6	7	0.007^	33.3	2	1.000^	100	6	0.350^
Thyroid gland	1.9	9	2.5	8	0.7	1	0.163*	50.0	4	0.448^	87.5	7	1.000^
Bladder	1.7	8	0.3	1	4.6	7	0.002^	0.0	0	0.057^	75.0	6	0.679^
Kidney	1.3	6	0.3	1	3.3	5	0.016^	40.0	2	0.665^	100	5	0.589^
Liver	1.1	5	0.6	2	2.0	3	0.336^	50.0	2	0.600^	100	5	0.589^
Esophagus	1.1	5	0.3	1	2.6	4	0.041^	0.0	0	0.177^	60.0	3	0.287*
Pancreas	0.6	3	0.3	1	1.3	2	0.250^	0.0	0	0.554^	66.7	2	0.511^
Gallbladder	0.6	3	0.6	2	0.7	1	1.000^	0.0	0	1.000^	66.7	2	0.511*
Neuroendocrine	0.4	2	0.6	2	0.0	0	1.000^	50.0	1	0.549^	100	2	1.000^
Other cancer sites	2.1	10	2.9	9	0.7	1	0.177^	30.0	3	1.000^	90.0	9	0.696*
**In active cancer treatment**							0.332*			0.302*			0.055*
Yes	33.8	158	35.2	111	30.7	47		36.0	54		84.0	126	
No	66.2	310	64.8	204	69.3	106		31.1	89		76.1	220	

^1^ CAM level 2: One or more visits to CAM providers; ^2^ CAM level 3: One or more visits to CAM providers, use of CAM natural remedies and/or use of CAM self-help practices; * Pearson chi-square test; ^Fisher exact test; **The cancer could be placed at more than one site

### Visits to CAM providers

Of the 468 participants, 436 replied to the questions regarding modalities offered by CAM providers. Of these 33% (*n* = 143) visited CAM providers to receive one or more of the modalities listed in Table 3 in the time after their first cancer diagnosis, 30% (*n* = 43) used more than one modality with a mean of 1.5 different provider-based CAM modalities (range 1–6). The most frequently used CAM modality was *massage/aromatherapy* used by 19% (*n* = 84) followed by *acupuncture* (11%, *n* = 48), *osteopathy* (4%, *n* = 18), *naprapathy* (4%, *n* = 18), and *healing* (4%, *n* = 17). Most participants visited CAM providers for well-being and to improve quality of life (64%, *n* = 91) or to treat adverse effects/late and long-term effects of their cancer/cancer treatment (59%, *n* = 85). Only 10 participants (7%) had used the modalities to treat the cancer or prevent it from spreading; *healing* (*n* = 5), *herbal therapy* (*n* = 2), *acupuncture* (*n* = 2) and *homeopathy* (*n* = 1). Very few (8%, *n* = 11) experienced adverse effects after seeing a CAM provider, mainly from *acupuncture* (*n* = 5; 4 mild and 1 moderate), and *massage* (*n* = 3; 1 mild and 2 moderate, Table 3).

**Table 3:** Provider-based CAM modalities used by cancer patients, reason(s) for use, and adverse effects of treatment

					**Reason(s) for use** (multiple choice)					
	**Total**	**Women**	**Men**	***p*-value**	**To treat cancer or prevent it from spreading**	**To treat side effects or late effects of cancer/ cancer treatment**	**To strengthen the body / immune system**	**To increase quality of life, for coping, relaxation or well-being**	**Other reasons**	**Adverse effects of treatment**
	% (n)	% (n)	% (n)		% (n)	% (n)	% (n)	% (n)	% (n)	% (n)
Massage/ aromatherapy	19.3 (84)	23.9 (70)	9.8 (14)	< 0.001*	0.0 (0)	56.0 (47)	22.6 (19)	78.6 (66)	0.0 (0)	3.6 (3)
Acupuncture	11.0 (48)	13.3 (39)	6.3 (9)	0.028*	4.2 (2)	70.8 (34)	29.2 (14)	47.9 (23)	12.5 (6)	10.4 (5)
Naprapathy	4.1 (18)	3.8 (11)	4.9 (7)	0.574*	0.0 (0)	55.6 (10)	33.3 (6)	55.6 (10)	33.3 (6)	11.1 (2)
Osteopathy	4.1 (18)	5.8 (17)	0.7 (1)	0.012*	0.0 (0)	83.3 (15)	22.2 (4)	44.4 (8)	16.7 (3)	0.0 (0)
Healing	3.9 (17)	4.4 (13)	2.8 (4)	0.406*	29.4 (5)	23.5 (4)	17.6 (3)	67.7 (11)	5.9 (1)	5.9 (1)
Reflexology	2.3 (10)	3.4 (10)	0.0 (0)	0.035^	0.0 (0)	50.0 (5)	80.0 (8)	60.0 (6)	20.0 (2)	10.0 (1)
Coaching	2.3 (10)	3.1 (9)	0.7 (1)	0.177^	0.0 (0)	10.0 (1)	0.0 (0)	100 (10)	10.0 (1)	10.0 (1)
Homeopathy	1.8 (8)	2.7 (8)	0.0 (0)	0.057^	12.5 (1)	50.0 (4)	50.0 (4)	25.0 (2)	37.5 (2)	12.5 (1)
Herbal therapy	0.9 (4)	1.4 (4)	0.0 (0)	0.309^	50.0 (2)	75.0 (3)	75.0 (3)	50.0 (2)	0.0 (0)	25.0 (1)
Rosen therapy	0.2 (1)	0.3 (1)	0.0 (0)	1.000^	0.0 (0)	0.0 (0)	0.0 (0)	100 (1)	0.0 (0)	0.0 (0)
Other provider-based therapies^1^	17.5 (75)	20.6 (59)	11.3 (16)	0.016*	-	-	-	-	-	-
**Consultations with CAM providers**	**32.8 (143)**	**38.9 (114)**	**20.3 (29)**	< 0.001*	**7.0 (10)**	**59.4 (85)**	**30.1 (43)**	**63.6 (91)**	**9.1 (13)**	**7.7 (11)**

^*^Pearson Chi-square test; ^Fisher exact test; ^1^Not included in overall CAM due to uncertainty of this being CAM;—not collected

Most of the participants experienced the treatments as beneficial (87%, *n* = 125), and none experienced worsening of symptoms due to the treatments. Forty-three percent of the participants obtained information about provider-based CAM from health care providers (43%, *n* = 62), followed by family/friends (34%, *n* = 49), the internet/media (25%, *n* = 36), or from CAM providers (13%, *n* = 19). Fourteen percent (*n* = 20) consulted other sources while 7% (*n* = 10) did not obtain information about the modalities they used. With regard to discussing the visits to CAM providers with health care providers, 46% (*n* = 66) reported that they had discussed it with their GP, 30% (*n* = 43) with their oncologist, 13% (*n* = 18) with a nurse, 8% (*n* = 11) with a CAM provider while 19% (*n* = 27) had discussed the use with other health care providers. Thirty-two percent (*n* = 45) had not discussed this with any of the above-mentioned providers. Multiple answers were possible for information and communication (Table 4).

**Table 4:** Self-reported effect, information and disclosure of CAM use

	**CAM provider**	**Natural remedies**	**Self-help practices**	**Special diets**	**Physical activity**	**Spiritual practices**
	% (*n* = 143)	% (*n* = 230)	% (*n* = 253)	% (*n* = 13)	% (*n* = 405)	% (*n* = 132)
Self-reported effect*						
Better	87.4 (125)	34.5 (79)	80.6 (204)	46.2 (2)	83.1 (325)	28.9 (37)
No change	7.7 (11)	41.5 (95)	10.3 (26)	7.7 (1)	10.0 (39)	45.3 (58)
Worse	0.0 (0)	0.0 (0)	0.0 (0)	0.0 (0)	1.0 (4)	0.0 (0)
Don't know	4.9 (7)	24.0 (55)	9.1 (23)	46.2 (6)	5.9 (23)	25.8 (33)
Information**						
Internet / media	25.2 (36)	45.7 (105)	34.4 (87)	61.5 (8)	23.7 (96)	0.9 (4)
Health care providers	43.4 (62)	19.6 (45)	38.3 (97)	7.7 (1)	39.0 (158)	0.8 (1)
CAM providers	13.3 (19)	7.4 (17)	6.3 (16)	23.1 (3)	3.0 (12)	0.8 (1)
Friends, family	34.3 (49)	28.3 (65)	28.5 (72)	38.5 (5)	24.7 (100)	29.5 (39)
Other	14.0 (20)	18.3 (42)	20.6 (52)	15.4 (2)	19.0 (77)	21.2 (28)
Do not remember	5.6 (8)	5.2 (12)	6.3 (16)	0.0 (0)	7.4 (30)	3.0 (4)
Did not seek/receive	7.0 (10)	12.6 (29)	15.4 (39)	0.0 (0)	24.7 (100)	43.9 (58)
Communication**						
General practitioner	46.2 (66)	21.3 (49)	32.8 (83)	7.7 (1)	41.2 (167)	0.8 (1)
Oncologist	30.1 (43)	17.0 (39)	24.5 (62)	38.5 (5)	29.1 (118)	0.0 (0)
Nurse	12.6 (18)	5.7 (13)	16.2 (41)	7.7 (1)	14.8 (60)	1.5 (2)
CAM provider	7.7 (11)	10.9 (25)	16.6 (42)	23.1 (3)	0.0 (0)	0.8 (1)
Other health care providers	18.9 (27)	5.7 (13)	5.9 (15)	23.1 (3)	17.3 (70)	0.8 (1)
None of these	31.5 (45)	55.2 (127)	41.1 (104)	46.2 (6)	32.6 (132)	88.6 (117)
Do not remember	3.5 (5)	4.8 (11)	4.3 (11)	0.0 (0)	5.9 (24)	4.5 (6)

^*^Due to missing responses the sum of the numbers does not always add up to the total number; **Multiple choice

### Use of natural remedies

Of the 468 participants, 441 replied to the questions regarding natural remedies. Of these 52% (*n* = 230) report to have used one or more of the natural remedies listed in Table 5 with 60% (*n* = 138) using more than one remedy with a mean of 2.4 remedies used (range 1–10). The most frequently used remedy was *Omega 3, 6, 9 fatty acids* (31%, *n* = 138) followed by *ginger* (20%, *n* = 86), *green tea,* and *blueberries/blueberry extract* (both 17%, *n* = 74). Most of the natural remedies were used to strengthen the body or the immune system (90%, *n* = 207) while 39% (*n* = 90) used it with the intention to increase the quality of life, coping, relaxation or well-being. However, 20% use it to treat the cancer or prevent it from spreading and 24% used it to manage adverse effects/late and long-term effects of cancer/ cancer treatment. Few (6%, *n* = 17) experienced adverse effects from natural remedies, mainly from *Omega 3, 6, 9 fatty acids* (5 mild and 1 moderate, Table 5).

**Table 5:** Natural remedies used by cancer patients, reason(s) for use, and adverse effects of treatment

					**Reason(s) for use** (multiple choice)					
	**Total**	**Women**	**Men**	***p*-value**	**To treat cancer or prevent it from spreading**	**To treat side effects or late effects of cancer/ cancer treatment**	**To strengthen the body / immune system**	**To increase quality of life, for coping, relaxation or well-being**	**Other reasons**	**Adverse effects of treatment**
	% (n)	% (n)	% (n)		% (n)	% (n)	% (n)	% (n)	% (n)	% (n)
Omega 3, 6, 9 fatty acids	31.3 (138)	31.3 (93)	31.3 (45)	0.937*	8.0 (11)	18.8 (26)	90.6 (125)	26.1 (36)	2.2 (3)	4.3 (6)
Ginger	19.5 (86)	23.2 (69)	11.8 (17)	0.007*	16.3 (14)	20.9 (18)	80.2 (69)	34.9 (30)	12.8 (11)	1.2 (1)
Green tea	16.8 (74)	19.5 (58)	11.1 (16)	0.040*	17.6 (13)	13.5 (10)	79.2 (59)	51.4 (38)	4.1 (3)	4.1 (3)
Blueberries / blueberry extract	16.8 (74)	18.5 (55)	13.2 (19)	0.169*	14.9 (11)	12.2 (9)	97.3 (72)	23.0 (17)	6.6 (5)	4.1 (3)
Garlic	15.2 (67)	13.8 (41)	18.1 (26)	0.231*	17.9 (12)	13.4 (9)	89.6 (60)	28.4 (19)	10.4 (7)	3.0 (2)
Turmeric / curcumin	11.4 (50)	12.8 (38)	8.3 (12)	0.201*	40.0 (20)	30.0 (15)	80.0 (40)	32.0 (16)	2.0 (1)	4.0 (2)
Aloe vera	3.9 (17)	3.7 (11)	4.2 (6)	0.807*	5.9 (1)	35.3 (6)	35.3 (6)	41.2 (7)	17.6 (3)	11.8 (2)
Chaga	3.2 (14)	4.4 (13)	0.7 (1)	0.043^	57.1 (8)	14.3 (2)	64.3 (9)	14.3 (2)	7.1 (1)	7.1 (1)
Echinacea	1.6 (7)	2.0 (6)	0.7 (1)	0.435*	14.3 (1)	14.3 (1)	85.7 (6)	14.3 (1)	0.0 (0)	14.3 (1)
Q10	1.6 (7)	2.0 (6)	0.7 (1)	0.435*	0.0 (0)	42.9 (3)	85.7 (6)	28.6 (2)	14.3 (1)	14.3 (1)
Ginseng	0.9 (4)	1.0 (3)	0.7 (1)	0.605^	25.0 (1)	25.0 (1)	100 (4)	50.0 (2)	0.0 (0)	25.0 (1)
Medicinal mushrooms (Reishi, Maitake, Shiitake)	0.7 (3)	0.7 (2)	0.7 (1)	1.000^	66.7 (2)	33.3 (1)	100 (3)	33.3 (1)	0.0 (0)	0.0 (0)
Cannabis	0.7 (3)	0.3 (1)	1.4 (2)	0.250^	0.0 (0)	0.0 (0)	33.3 (1)	33.3 (1)	33.3 (1)	0.0 (0)
Noni-juice	0.7 (3)	0.7 (2)	0.7 (1)	1.000^	33.3 (1)	66.7 (2)	100 (3)	0.0 (0)	0.0 (0)	0.0 (0)
Birch sap	0.5 (2)	0.3 (1)	0.7 (1)	0.545^						
Mistletoe/Iscador	0.5 (2)	0.7 (2)	0.0 (0)	0.455^	100 (2)	0.0 (0)	50.0 (1)	0.0 (0)	0.0 (0)	50.0 (1)
Evening primrose oil	0.5 (2)	0.7 (2)	0.0 (0)	0.455^	50.0 (1)	0.0 (0)	0.0 (0)	50.0 (1)	0.0 (0)	0.0 (0)
Rosenrot	0.5 (2)	0.7 (2)	0.0 (0)	0.452^	0.0 (0)	50.0 (1)	50.0 (1)	100 (2)	0.0 (0)	0.0 (0)
Shark cartilage	0.2 (1)	0.3 (1)	0.0 (0)	0.673^	0.0 (0)	100 (1)	100 (1)	0.0 (0)	0.0 (0)	0.0 (0)
Milk thistle	0.0 (0)	0.0 (0)	0.0 (0)	-	-	-	-	-	-	-
Other natural remedies^1^	6.8 (30)	8.4 (25)	3.5 (5)	0.084*	-	-	-	-	-	-
**Use of natural remedies**	**52.2 (230)**	**53.5 (159)**	**49.3 (71)**	**0.418**	**20.0 (46)**	**23.9 (55)**	**90.0 (207)**	**39.1 (90)**	**12.2 (28)**	**6.1 (17)**

^*^ Pearson Chi-square test; ^Fisher exact test; ^1^Not included in overall natural remedies due to uncertainty of this being CAM; - not collected

About a third of the participants experienced that the remedies were beneficial for them (35%, *n* = 79), and 42% (*n* = 95) did not experience any change due to the natural remedies. None experienced worsening of their symptoms due to the remedies (Table 4).

Almost half of the participants (46%, *n* = 105) gathered information about natural remedies from the internet or media while 28% (*n* = 65) sought or received information from family and friends. Twenty percent (*n* = 45) obtained information from health care providers and 7% (*n* = 17) from CAM providers. Eighteen percent (*n* = 42) used other sources and 13% (*n* = 29) did not obtain information. A total of 21% (*n *= 49) disclosed the use of natural remedies to their GP, 17% (*n* = 39) to their oncologist, 6% (*n* = 13) to a nurse; 11% (*n* = 25) to a CAM provider while 6% (*n* = 13) discussed the use with other health care providers. More than half of the users of natural remedies (55%, *n* = 127) did not disclose their use to any of the providers mentioned above (Table 4).

### Self-help practices

Of the 468 participants, 437 replied to the questions regarding self-help practices. Of these, 58% (*n* = 253) report to have used one or more of the self-help practices listed in Table 6. More than one self-help practice was used by 66% (*n* = 166) with a mean of 2.2 self-help practices used (range 1–6). Almost half of the participants (49%, *n *= 213) used *relaxation techniques*, followed by *meditation* (29%, *n* = 127), and *yoga* (28%, *n* = 122), mostly to increase quality of life (94%, *n* = 200, *n* = 119, and *n* = 115 respectively). Few people experienced adverse effects from self-help practices (6%, *n* = 16, Table 6), mostly from *relaxation* *techniques *(*n* = 11), *meditation* (*n* = 8), and *yoga* (*n *= 7). Most of the adverse effects were mild or moderate but two were reported to be severe, one from *yoga* and one from *art therapy*. The majority (81%, *n* = 204) found the practices helpful (Table 4). None experienced worsening of their symptoms. One-third of the participants obtained information on the self-help practices from health care providers (38%, *n* = 97), followed by internet/media (34%, *n* = 87), and friends and family (29%, *n* = 72). Few obtained information from CAM providers (6%, *n* = 16). Fifteen percent (*n* = 39) did not seek or receive information about the practices used (Table 4). With regard to discussing self-help practices with health care providers, 33% (*n* = 83) reported that they had discussed it with their GP, 25% (*n *= 62) with their oncologist, 16% (*n* = 41) with a nurse, 17% (*n* = 42) with a CAM provider while 6% (*n* = 15) had discussed the practices with other health care providers. Forty-one percent (*n* = 104) had not discussed this with any of the above-mentioned providers (Table 4).

**Table 6:** Self-help practices used by cancer patients, reason(s) for use, and adverse effects of treatment

					**Reason(s) for use** (multiple choice)					
	**Total**	**Women**	**Men**	***p*-value**	**To treat cancer or prevent it from spreading**	**To treat side effects or late effects of cancer/ cancer treatment**	**To strengthen the body / immune system**	**To increase quality of life, for coping, relaxation or well-being**	**Other reasons**	**Adverse effects of treatment**
	% (*n*)	% (n)	% (n)		% (n)	% (n)	% (n)	% (n)	% (n)	% (n)
Relaxation	48.7 (213)	55.8 (164)	34.3 (49)	< 0.001*	5.2 (11)	33.8 (72)	36.6 (78)	93.9 (200)	1.4 (3)	5.2 (11)
Meditation/mindfulness	29.1 (127)	38.1 (112)	10.5 (15)	< 0.001*	7.1 (9)	38.6 (49)	39.4 (50)	93.7 (119)	4.7 (6)	5.5 (8)
Yoga	27.9 (122)	38.1 (112)	7.0 (10)	< 0.001*	4.1 (5)	45.1 (55)	57.4 (70)	94.3 (115)	5.7 (7)	5.7 (7)
Visualization	7.1 (31)	9.2 (27)	2.8 (4)	0.015*	9.6 (3)	22.6 (7)	35.5 (11)	93.5 (29)	9.7 (3)	6.5 (2)
Music therapy	5.0 (22)	4.4 (13)	6.3 (9)	0.410*	0.0 (0)	18.2 (4)	9.1 (2)	95.5 (21)	13.6 (3)	4.5 (1)
Tai chi / qigong	4.1 (18)	5.4 (16)	1.4 (2)	0.046*	5.6 (1)	33.3 (6)	61.1 (11)	94.4 (17)	0.0 (0)	11.1 (2)
Art therapy	2.3 (10)	3.4 (10)	0.0 (0)	0.035^	0.0 (0)	30.0 (3)	20.0 (2)	90.0 (9)	20.0 (2)	1.0 (1)
Astrology/numerology/fortuneteller	0.9 (4)	1.4 (4)	0.0 (0)	0.308^	0.0 (0)	0.0 (0)	0.0 (0)	75.0 (3)	25.0 (1)	0.0 (0)
Other self-help practices^1^	26.1 (114)	27.9 (82)	22.4 (32)	0.210*	-	-	-	-	-	-
**Self-help practices**	**57.9 (253)**	**66.3 (195)**	**40.6 (58)**	<0.001*	**6.3 (16)**	**39.9 (101)**	**46.2 (117)**	**96.0 (243)**	**5.9 (15)**	**6.3 (16)**

^*^ Pearson Chi-square test; ^Fisher exact test; ^1^not included in overall CAM self-help practices due to uncertainty of this being CAM;—not collected

### Special diets

Very few participants (3%, *n *= 13) had used special diets, only 5 men and 8 women (*p* = 0.766). Two different diets were reported; *Juice diet* (2%, *n* = 8) and *Budwig diet* (a diet consisting of a special lacto-vegetarian regimen with a blend of oil and protein [27], 1%, *n* = 6). All but one participant had used only one special diet (86%) leading to a mean of 1.1 diets used (range 1–2). These diets were mainly used to treat the cancer or prevent it from spreading (85%, *n* = 11) or to strengthen the body and the immune system (77%, *n* = 10). Two people experienced improvements after using the diets, and nobody experienced worsening of their symptoms (Table 4). However, 4 out of 8 participants (50%) experienced adverse effects from the *juice diet*: 1 moderate and 3 mild adverse effects were reported (Table 7). Most of the participants who had used special diets found the information about these diets on the internet or in the media (62%, *n* = 8), and 54% had discussed the use with health care providers, mostly their oncologist (39%, *n* = 5, Table 4).

**Table 7:** Special diets, physical activity and spiritual practices used by cancer patients, reason(s) for use, and adverse effects of treatment

					**Reason(s) for use** (multiple choice)					
	**Total**	**Women**	**Men**	**p-value**	**To treat cancer or prevent it from spreading**	**To treat side effects or late effects of cancer/ cancer treatment**	**To strengthen the body / immune system**	**To increase quality of life, for coping, relaxation or well-being**	**Other reasons**	**Adverse effects of treatment**
	% (n)	% (n)	% (n)		% (n)	% (n)	% (n)	% (n)	% (n)	% (n)
**Special diets**	**2.9 (13)**	**2.7 (8)**	**3.4 (5)**	**0.766^**	**84.6 (11)**	**38.4 (5)**	**76.9 (10)**	**38.4 (5)**	**0.0 (0)**	**30.8 (4)**
Juice diet (carrot, beetroot, apricot etc.)	1.8 (8)	1.7 (5)	2.0 (3)	0.723^	62.5 (5)	50.0 (4)	87.5 (7)	50.0 (4)	0.0 (0)	50.0 (4)
Breuss diet	0.0 (0)	0.0 (0)	0.0 (0)	-	-	-	-	-	-	-
Budwig diet	1.3 (6)	1.3 (4)	1.4 (2)	1.000^	100 (6)	16.6 (1)	66.7 (4)	16.6 (1)	0.0 (0)	0.0 (0)
Gerson therapy	0.0 (0)	0.0 (0)	0.0 (0)	-	-	-	-	-	-	-
Macrobiotic diet	0.0 (0)	0.0 (0)	0.0 (0)	-	-	-	-	-	-	-
Ornish diet	0.0 (0)	0.0 (0)	0.0 (0)	-	-	-	-	-	-	-
**Physical activity**	**93.3 (405)**	**92.8 (270)**	**94.4 (135)**	0.525*	**8.9 (36)**	**44.9 (182)**	**73.6 (298)**	**91.6 (372)**	**8.9 (36)**	**11.4 (46)**
Walks in the nature	84.3 (366)	84.9 (247)	83.2 (119)	0.591*	7.1 (26)	37.2 (136)	69.1 (253)	92.9 (340)	3.0 (11)	-
Walks along the road	74.9 (325)	76.6 (222)	72.0 (103)	0.360*	5.5 (18)	35.7 (116)	64.9 (211)	78.8 (256)	5.5 (18)	-
Gym	42.4 (184)	43.0 (125)	41.3 (59)	0.737*	8.7 (16)	45.1 (83)	78.3 (144)	83.7 (154)	4.9 (9)	-
Customized training program	41.5 (180)	45.4 (132)	33.6 (48)	0.016*	6.7 (12)	51.1 (92)	78.9 (142)	87.2 (157)	5.6 (10)	-
Skiing (cross country, slalom)	26.3 (114)	26.5 (77)	25.9 (37)	0.848*	9.6 (11)	39.4 (45)	71.1 (81)	94.7 (108)	8.8 (10)	-
Jogging/running	21.2 (92)	21.0 (61)	21.7 (31)	0.836*	12.0 (11)	47.8 (44)	84.8 (78)	93.5 (86)	5.4 (5)	-
Ball games (e.g. football, handball)	1.6 (7)	1.0 (3)	2.8 (4)	0.224^	0.0 (0)	14.3 (1)	85.7 (6)	100 (7)	14.3 (1)	-
Other physical activity	48.2 (209)	50.2 (146)	44.1 (63)	0.240*	-	-	-	-	-	-
**Spiritual practices**	**30.4 (132)**	**32.3 (94)**	**26.6 (38)**	0.267*	**31.8 (42)**	**12.9 (17)**	**9.8 (13)**	**44.7 (59)**	**42.4 (56)**	**1.6 (2)**
Prayer for one-self	19.6 (85)	20.6 (60)	17.5 (25)	0.429*	16.5 (14)	8.2 (7)	9.4 (8)	55.3 (47)	42.4 (36)	-
Prayed for by others	19.6 (85)	20.6 (60)	17.5 (25)	0.471*	42.4 (36)	16.5 (14)	8.2 (7)	25.9 (22)	37.6 (32)	-
Participation in religious assembly	3.9 (17)	3.4 (10)	4.9 (7)	0.470*	0.0 (0)	0.0 (0)	0.0 (0)	76.5 (13)	35.3 (6)	-
Contact with religious healer	0.7 (3)	0.3 (1)	1.4 (2)	0.254^	66.7 (2)	33.3 (1)	0.0 (0)	66.7 (2)	0.0 (0)	-
Shamanism	0.0 (0)	0.0 (0)	0.0 (0)	-	-	-	-	-	-	-
Other spiritual practices	3.2 (14)	3.1 (9)	3.5 (5)	0.782^	-	-	-	-	-	-

* Pearson Chi-square test; ^Fisher exact test;—not collected

### Physical activity

Most of the participants (93%, *n* = 405) were physically active of whom 95% (*n* = 383) were engaged in more than one activity with an average of 3.6 different physical activities (range 1–7). The rationale for the engaging in physical activities were mostly to increase the quality of life, cope with the illness, relax or improve well-being (92%, *n* = 372), or to strengthen the body and the immune system (74%, *n* = 298). The most frequent activities were *walks* (88%, *n* = 381), either in nature (84%, *n* = 366) or along the road (75%, *n* = 325), but also visits to *the gym* (42%, *n* = 184) and *customized training programs* (42%, *n* = 180) were popular activities (Table 7). Most of the participants found that these activities improved their health (83%. *n* = 325, Table 4). However, some (11%, *n* = 46, Table 7), reported adverse effects of their physical activity, mostly moderate (*n *= 23) and mild (*n* = 22), but also one (*n* = 1) severe. Information about physical activities was gathered from health care providers (39%, *n *= 158), friends and family (25%, *n* = 100) or sourced on the internet or in the media (24%, *n* = 96). Only 3% (*n* = 12) received information about physical activities from CAM providers. Twenty-five percent (*n* = 100) did neither receive nor seek information about physical activities (Table 4). Most of the participants (67%, *n* = 271) discussed their physical activities with health care providers, mostly with their GP (42%, *n* = 167), oncologist (29%, *n* = 118) or with a nurse (15%, *n *= 60), but also with other healthcare providers (17%, *n* = 70). Non discussed their physical activities with CAM providers (Table 4).

### Spiritual practices

One-third of the participants (30%, *n* = 132) reported to have participated in spiritual practices, 40% (*n* = 53) thereof engaged in more than one practice with a mean of 1.5 different spiritual practices (range 1–4). *Prayer* was the most practiced. They prayed themselves (20%, *n* = 85) or were prayed for by others (20%,*n* = 85). The majority prayed to increase quality of life, to cope, to relax, enhance well-being (45%, *n* = 59), or other reasons (42%, *n* = 56). Two people experienced adverse effects of spiritual practices (prayer), one moderate and one mild (Table 7). A total of 29% (*n* = 37) reported improvement from these spiritual practices, while 45%, (*n* = 58) reported no change (Table 4). Mostly they did not seek nor receive information about their spiritual practices (44%, *n* = 58) but 30% (*n* = 39) obtained information from family or friends. Some also sought information from other sources (21%, *n *= 28). The spiritual practices were mainly not discussed with health care providers (89%, *n* = 117, Table 4).

### Use of CAM in accordance with the NAFKAM model of reporting CAM

When reported CAM use was adapted to the NAFKAM model of reporting CAM, we found that 33% (*n* = 143) reported CAM at level 2, 79% at level 3 and 4 (*n* = 346 and 347 respectively), 96% (*n* = 421) at level 5 and 97% (*n* = 424) at level 6. CAM was more frequently used by women than men at CAM level 2–4. For CAM levels 5 and 6 no gender differences were found (Table 8).

**Table 8:** Level-differentiated use of CAM, reason(s) for use and adverse effects of treatment

					Reason(s) for use (multiple choice)						
	Total	Women	Men	*p*-value*	To treat cancer or prevent it from spreading	To treat side effects or late effects of cancer/ cancer treatment	To strengthen the body / immune system	To increase quality of life, coping, relaxation or well-being	Other reasons	Adverse effects	
	% (n)	% (n)	% (n)	% (n)	% (n)	% (n)	% (n)	% (n)	% (n)	% (n)	
**CAM level 2**	32.8 (143)	38.9 (114)	20.3 (29)	<0.001	7 (10)	59.4 (85)	30.1 (43)	63.6 (91)	9.1 (13)	7.7 (11)	
**CAM level 3**	78.8 (346)	83.1 (246)	69.9 (100)	0.002	15.9 (55)	48.0 (166)	73.1 (253)	79.2 (274)	13.6 (47)	9.0 (31)	
**CAM level 4**	79.0 (347)	83.1 (246)	70.6 (101)	0.003	16.4 (57)	48.1 (167)	73.2 (254)	79.3 (275)	13.5 (47)	9.8 (34)	
**CAM level 5**	96.1 (421)	96.6 (285)	95.1 (136)	0.444	16.9 (71)	55.8 (235)	82.7 (348)	93.8 (395)	16.2 (68)	15.2 (64)	
**CAM level 6**	96.8 (424)	96.9 (286)	96.5 (138)	0.804	23.6 (100)	56.6 (240)	82.3 (349)	93.6 (397)	25.7 (109)	15.1 (64)	

*Pearson chi-square test; CAM level 2: One or more visits to CAM providers; CAM level 3: CAM level 2 and/or use of natural remedies and self-help practices; CAM level 4: CAM level 3 and/or of special diets; CAM level 5: CAM level 4 and/or use of physical activity; CAM level 6: CAM level 5 and/or use of spiritual practices

The most common reason for CAM use was to increase the quality of life for all levels of CAM (79%-94%). The highest use of CAM for treating the cancer or preventing it from spreading was found at level 6 where spiritual practices were added (Tables 7 and 8). Adverse effects were few at all levels (8%-15%), highest at level 5 where physical activity was added to visits to CAM providers, use of natural remedies, self-help practices, and special diets (Table 7).

## Discussion

### Main findings

A large proportion of cancer patients included in this survey (79%) had used CAM (level 3); 33% had seen CAM providers, 52% had used natural remedies while 58% had employed self-help practices. The cancer patients primarily used the different CAM modalities to increase the quality of life, cope with the cancer disease, or for relaxation/well-being (64%-94%). Participants experienced high satisfaction with visits to CAM providers and self-help practices in terms of improvement of symptoms (87% and 81% respectively); however, not to the same degree for use of natural remedies (35%). Only few reported adverse effects of their CAM treatments (9%). Many users had multiple reasons/motives for using a CAM modality. For information about CAM modalities, participants most often searched the internet for natural remedies (45%), while healthcare providers were consulted for information about provider based CAM therapies (43%) and self-help practices (38%). A total of 41% did not discuss their use of CAM with a physician.

### Agreements and disagreements with other studies

Prevalence of CAM use at level 3 (CAM provider, natural remedies, and/or self-help practices) among Norwegian cancer patients found in this study was higher than what has previously been reported among cancer patients in Scandinavia in general (36%) [28] and in several Nordic studies [7, 8, 29, 30]. A Swedish study found that 26% of the cancer patients had used CAM at level 4 [8] after being diagnosed with cancer. A Danish study among breast cancer patients reported 50% use of CAM [29] while a Danish study among colorectal cancer patients reported 49%, both at level 3 [30]. A previous Norwegian study among cancer patients found 33% use of CAM at level 3, all three studies (the two Danish and the previous Norwegian study) within a time frame of 12 months [7]. It was also higher than what was found in studies from Europe (30%) [14], North America (46%), Australia/New Zealand (40%) [9], and in a recent worldwide systematic review (51%) [10]. One reason for this discrepancy in prevalence may be the large number of CAM modalities specified in our study. The specified modalities served as a reminder for participants and informed them how to define CAM. The CAM use reported in our study is in line with findings from other studies using equally specified questionnaires, such as a German study in which 78% of breast cancer patients reported to have used CAM (at level 3) in the previous 12 months [31]. Breast cancer patients are known to be more frequent users of CAM than patients with other cancer diagnoses [10, 31, 32]. This factor may also contribute to the high prevalence of CAM use in our study as 39% of the participants suffered from breast cancer. Also, the high percentage of middle-aged, university-educated women in the study can have contributed to the high number of participants reporting to have used CAM as both female gender, higher education, and young to middle-age are prediction of CAM use. The category “time since diagnosis” might also influence the high prevalence of use in our study as we included responses from participants who had been diagnosed with cancer many years ago. Several of the above-mentioned studies reported CAM use within a time frame of 12 months or during cancer treatment while we asked for use since diagnosed with cancer.

The fact that the study was conducted 1½ years into the COVID-19 pandemic might also have influenced the results as people seem to use more CAM during the pandemic than before, especially self-administered modalities like self-help techniques and natural remedies [33]. This is in line with this present study where self-administrated modalities were most frequently used. The high use of self-administrated modalities is in accordance with findings from other studies [8], where natural remedies were frequently used [7, 34]. The most commonly used self-administrated modality in the present study was relaxation therapy (49%), which reflects the reported main reasons for overall CAM use to increase the quality of life, cope, relax and improve well-being. These reasons for CAM use are in accordance with findings from the Swedish study [8] where the main reason for CAM use was to increase well-being. According to research [35], stress may have a negative influence on cancer patients and their immune response, and potentially interfere in the development and progression of the disease. Relaxation therapies have demonstrated to be beneficial for reducing stress in cancer patients [35].

The second most used CAM modality in this study was Omega 3, 6, or 9 fatty acids (31%). The relationship between omega 3, 6 or 9 fatty acids and cancer risk is unclear [36, 37]. We found; however, that the participants used these fatty acids (91%) to strengthen the body and the immune system. Only 8% used it to treat the cancer or prevent it from spreading. Twenty-six percent used it to increase the quality of life. This was in particular true for the participants who suffered from depression and anxiety as a consequence of their cancer. Depression influences the quality of life of around 20% of cancer patients and antidepressants are the most well-established treatment for depression in cancer alongside different psychotherapeutic interventions. Many patients experience; however, adverse effects of antidepressant medication [38], while at the same time the access to psychotherapeutic interventions are limited [39]. Thus, an accessible intervention with fewer adverse effects is needed for the management of depression in cancer patients. Psychiatry studies investigated the association between depression and omega-3 fatty acid as a potential complementary and well-tolerated intervention for cancer patients suffering from depression [38]. Several meta-analyses have reported positive outcomes for omega-3 fatty acids in the treatment of depression [40–43], although a Cochrane review concluded that overall results are not unanimously positive [44].

Although the majority of users did not use natural remedies with the intention to treat or prevent cancer, it is interesting to note that the use of some of these specific remedies (e.g. omega-3,6, 9, ginger, green tea, and garlic) are remarkably more frequently used compared to what has recently been reported by our research group in a general population-based study using the I-CAM-Q questionnaire [33]. This higher usage among persons with cancer or with a history of cancer compared to the general population confirms the particular need for good information and communication strategies of CAM in the context of cancer care [45].

The modalities most frequently used to treat cancer/prevent it from spreading were turmeric / curcumin (n = 20), ginger (n = 13), and green tea (n = 13). The minority of the participants; however, applied these modalities, leading to an overall use of CAM (level 3) of 16% (n = 55) to treat the cancer or prevent it from spreading. This is somewhat lower than what was found in a recent systematic review where to treat or cure cancer was found to be the most common reason for CAM use. A reason for this discrepancy might be the legal situation in Norway where CAM providers are not allowed to treat the cancer disease unless this is done in accordance with the patient’s physician or no curative or palliative treatment is available for the patient [10]. Only treatment with the purpose to manage consequences of the disease or treatment-related adverse effects, or to strengthen the body’s immune system and its ability to heal itself is otherwise allowed for CAM providers [10].

Our finding of female cancer patients using more CAM at levels 2–4 is in line with the majority of other studies, both nationally [7, 46, 47] and internationally [48–51]. Women use health care services, in general, more frequently than men [15, 52–55] but report to have more unmet health care needs within conventional health care than men [52]. This may be the reason why women choose to use CAM to a higher degree than men [7, 56, 57].

### Information

In contrast to earlier findings showing that CAM users primarily obtain information about CAM modalities on the internet, in the media, and among friends and family [58]; half of the CAM users (at level 3) in the present study obtain information about CAM from health care providers, internet and media (47%), and family and friends (39%). These results corroborate previous studies reporting that patients prefer to receive information about CAM from their healthcare providers [58, 59]. The findings are also in line with earlier findings showing that 50% of physicians and 57% of nurses in cancer care search for evidence-based information about CAM [60], presumably to pass it on to patients. The information might have been provided to the patient upon request when discussing their CAM use and not necessarily been offered routinely. Cancer patients require information about safety and efficacy from trustworthy sources and would appreciate a hospital-based CAM education program [58]. The majority of physicians (89%) and nurses (88%) in cancer care in Norway report to be moderately or very comfortable with answering questions about CAM [23]. NAFKAM has not only recognized the reported information need but also addressed it by publishing a specialist database of evidence-based information on CAM for cancer aimed at healthcare providers in English [61] as well as patient versions on its website in Norwegian [62]. Furthermore, NAFKAM and the NCS conducted 16 public theme meetings around the country, and a digital toolbox for health care providers on CAM has been created [63].

### Communication

Despite the fact that only 31% of Norwegian physicians in cancer care ask their patients about their CAM use on a regular basis [64], more than half of the CAM users (59%, level 3) in this study discussed one or more of the modalities they used with a physician, either their GP (49%) or their oncologist (36%). This is in accordance with a systematic review reporting non-disclosure rates of 20–77% with an average of 40–50% based on 21 international studies [65], and a previous Norwegian study among cancer patients receiving chemotherapy reporting non-disclosure rates of 28–54% [34]. While the disclosure rate in the present Norwegian study was well in line with internationally reported studies, the disclosure rate was higher than what was found in studies conducted in other Scandinavian countries [8, 30] yet lower than what was found in an American study among breast cancer patients (disclosure rates of 71- 85%) [66]. Reasons for disclosure or non-disclosure of CAM use can be diverse.

The *higher* disclosure rate in the present study compared to a Swedish study (disclosure rate of 33%) [8] and a Danish study with colorectal cancer patients (disclosure rate of 49%) might be due to the somewhat higher percentage of female physicians in Norway (54%) [67] compared to Sweden (44%) [68] and Denmark (51%) [69] as female physicians have been reported to discuss their patients’ use of CAM more often than their male colleagues [70]. In general female physicians seem to provide the patients with more patient-centred consultations [71] and spend more time with their patients [72], enabling the patients more time to raise the topic themselves. Although gender equality is high in all the Scandinavian countries, the percentage of female physicians is highest in Norway [67–69]. Furthermore, the current study asked about a wide range of CAM modalities and the majority of the participants (75%) used more than one CAM modality (range 1–17) with an average of 3.8 modalities each. Users might have only discussed the use of one of the modalities rather than all modalities, which might have led to a wider range of CAM modalities *not* discussed with physicians than the disclosure rate of 59% indicate. Disclosure of natural remedies was particularly low as only 30% reported discussing such use with a physician. This is in line with earlier findings showing that disclosure of CAM use to medical providers is lower for self-care than provider-based CAM [73]. The reason for the particularly low disclosure rate for natural remedies might be due to the fact that physicians often discourage such use [18, 74] because the potential risk of interactions with conventional cancer treatment is highest with these remedies.

The *lower* disclosure rates reported in the present study compared to those in the US study in breast cancer patients (disclosure rates of 71- 85%) [66] could be due to the fact that women in general are more likely to disclose their use of CAM [75, 76] and differences in consultation practices between Norway and the US. While integrative oncology is more common in the US, where also evidence-based guidelines for integrative oncology are available [77], it is rarely practiced in Norway [78]. Patients in Norway thus tend to administrate CAM and conventional care in a parallel manner [78, 79]. This might have contributed to a consultation practice where communication around the patient’s use of CAM does not have a natural place, resulting in lower disclosure rates compared to those reported in the US study [66].

Many patients do not disclose their CAM use simply because they are not asked or do not think this is of any relevance to the medical providers [56, 80] while others are afraid of being stigmatized if they disclose their use of CAM [80–83]. Disclosure is influenced by the nature of the patient-physician communication, and a belief in support for CAM use [84]. In some countries, the legal situation might also have an impact. In Norway, as mentioned above, are CAM providers only allowed to treat cancer patients for the sole purpose of managing side-effects from cancer and cancer treatment, or supporting the body’s immune system and ability to self-heal, unless the treatment is given in cooperation with the patient’s physician which is rare [85]. This may have led to nondisclosure of provider-based CAM treatment. This is not; however, true for the majority of the participants as only 7% reported provider-based CAM treatment aimed at treating cancer and prevent it from spreading.

Considering the high prevalence of CAM use and low disclosure rates it is paramount to support health literacy among patients. Oncology health care providers report a lack of knowledge as the most frequent reason for not asking patients about their use of CAM [45, 86]. To increase disclosure rates as well as to improve the quality of communication about CAM, a recent review and clinical practice guidelines has suggested seven clinical practice recommendations [45].

### Strengths and limitations of the study

The main strengths of the study are the rather high response rate, the adequate study power, the wide range of cancer sites and diagnoses, an age distribution similar to adult cancer survivors in Norway, and the geographical distribution of participants representing all parts of Norway, rural as well as urban. This study was not conducted in a hospital setting and thereby not limited to patients currently receiving conventional cancer treatment. The study must; however, be understood in light of some limitations. The main limitation of the study is that the members of the NCS’s user panel do not fully represent the total cancer population in Norway. For example, with respect to gender there were more female participants in the survey than female cancer patients in general (67% vs 46%). This bias was solved by partly presenting gender-specific data. Another limitation was that all groups had an option “Other therapies” without asking for specification. These options were excluded from the analyses because we could not determine whether they were CAM or not. This contributed, on the other hand, to a possible underreporting of CAM users.

### Implication of the findings

The high use of CAM among Norwegian cancer patients has several implications. Firstly, health care providers should routinely ask cancer patients about their CAM use. Many patients use herbs and other natural remedies, yet these can interact with conventional cancer treatment. Ginger (used by 20%), green tea (used by 17%), and turmeric / curcumin (used by 11%) are examples of herbs that can influence cancer and cancer treatment [87]. The risk of unwanted interactions increases when patients do not discuss such use with their oncologist, which only 17% of the users of natural remedies in this study did.

Secondly, considering that many CAM products are readily available over-the-counter or via the internet, and many patients choose self-care practices, there is also a particular need for improved health literacy among cancer patients. It is important that patients understand health-related information and can make informed decisions about their health, which includes discussions with their cancer care providers. NAFKAM and the NCS have worked closely to provide cancer patients with such information by organising information workshops with regional cancer groups, publishing patient information and general tools for understanding safety issues [62, 63, 88, 89] and will implement this new knowledge into future patient information.

Thirdly, the findings from this study will be included in teaching of health care providers and students, and in information to patients and relatives via various channels. The findings might further be relevant for advocacy work, e.g. via consultation input and the strategy of increased health competence in the population [90].

Finally, oncologists should also respond to patients’ unmet needs for supportive CAM treatment to improve quality of life and wellbeing as well as their desire to actively contribute to the treatment. The high satisfaction with several CAM modalities used to increase the quality of life reported in this study might be used to improve patient information of such modalities as earlier studies have revealed that patients value such information and prefer to receive it from health care providers [58, 59].

## Conclusion

Four out of five participants included in this study used CAM with high satisfaction and low rates of adverse effects. The main reasons for using CAM were to *increase quality of life, coping, relaxation or well-being* followed by *strengthening the body and the immune system*. Considering the high prevalence figures, reliable information provision supporting health care providers’ knowledge and health literacy among patients, as well as communication about benefits and harms of such treatments, are crucial. The cooperation between the NCS and NAFKAM provides an example of how to address these issues.

## Data Availability

The dataset this paper has been based on has not been deposited in any repository. All datasets and materials are available from the corresponding author upon reasonable request. Applicants for any data must, however be prepared to conform to Norwegian privacy regulations.

## Abbreviations

CAM: Complementary and Alternative Medicine
NCS: Norwegian Cancer Society
I-CAM-Q: International Questionnaire to Measure Use of Complementary and Alternative Medicine
GP: General practitioner
NAFKAM: National Research Center in Complementary and Alternative Medicine
NSD: Norwegian Centre for Research Data
